# Expansion of thyroid surgical territory through 10,000 cases under the da Vinci robotic knife

**DOI:** 10.1038/s41598-024-57163-2

**Published:** 2024-03-30

**Authors:** Jin Kyong Kim, Cho Rok Lee, Sang-Wook Kang, Jong Ju Jeong, Kee-Hyun Nam, Woong Youn Chung

**Affiliations:** 1 Department of Surgery, Severance Hospital, Yonsei Cancer Centre, Yonsei University College of Medicine, Seoul, 03722 South Korea; 2 Department of Surgery, Yongin Severance Hospital, Gyeonggi-do, South Korea

**Keywords:** Robotic transaxillary thyroid surgery, Thyroid cancer, Grave’s disease, Modern da Vinci robotic system, Thyroid cancer, Surgery, Therapeutic endoscopy

## Abstract

With the progress of robotic transaxillary thyroid surgery (RTTS), the indications for this procedure have gradually expanded. This study presents the insights gained from performing 10,000 RTTS cases at a single institution, along with the expansion of indications over time. RTTS was performed on 10,000 patients using the da Vinci robot system between October 2007 and April 2023 at the Yonsei University Health System, Seoul, Korea. Among 10,000 patients, 9461 (94.0%) were diagnosed with thyroid cancer, whereas 539 (5.4%) had either a benign thyroid nodule or Graves’ disease. Surgical procedures were performed using four-arm-based robots (da Vinci S, Si, or Xi) for 8408 cases (84.1%), with the remaining 1592 cases (15.9%) being performed using the da Vinci SP surgical robotic system. Notably, for 53 patients with nodules ≥ 5 cm, which were not included in the eligibility criteria of the previous study, RTTS was performed safely without significant complications. The most common postoperative complication was transient hypoparathyroidism (37.91%), and recurrence occurred in 100 patients with thyroid cancer (1.1%). In conclusion, RTTS appears safe and feasible from both surgical and oncological perspectives, and the spectrum of indications suitable for RTTS surgery is progressively expanding.

## Introduction

Since the first endoscopic parathyroidectomy, performed by Gagner^1^ in 1996, and the first robotic cholecystectomy, performed by Himpens and Cadière^2^ using the prototype of da Vinci surgical system in 1997, endoscopic surgery and robotic surgery now coexist as the two pillars of minimally invasive surgery. While both endoscopic and robotic instruments have been steadily advancing, in many countries, the choice of surgical approach is determined by the preference of the surgeon or patient.

In several regions, the modern da Vinci robotic system, originally designed for remote telesurgical procedures in battlefield settings^3^, has been extensively utilized for thyroid surgeries. Robotic surgery offers advantages such as three-dimensional imaging, motion scaling, tremor elimination, and additional degrees of freedom when compared with endoscopic surgery; however, many surgeons are proficient in performing laparoscopic surgery without the assistance of a robot.

Nevertheless, within the domain of thyroid surgery, the advantages of robotic surgery are maximized and surpass the limitations of endoscopic surgery^4^. Robotic or endoscopic thyroid surgery differs from other types of robotic surgery in terms of its nature and purpose. Most patients with thyroid cancer are young women who desire to conceal surgical scars as much as possible. This desire is strengthened by the favorable prognosis associated with most thyroid cancers. Various approaches have been developed for endoscopic surgery before robotic surgery, allowing surgeons to hide the scar through the cervical^5^, transaxillary^6,7^, bilateral axillary-breast^8^, retroauricular or facelift^9^, and transoral^10,11^ approaches.

Discomfort to the surgeons is unavoidable during endoscopic thyroid surgery^4,12,13^. This discomfort cannot be overcome by surpassing the learning curve or becoming familiar with the procedure. This is inherent to the nature of the surgery, as the target organ, the thyroid, is small and centrally located within the narrow cervical neck region. Numerous delicate structures, such as blood vessels, parathyroid glands, and important nerves, including the recurrent laryngeal nerve (RLN), vagus nerve, phrenic nerve, and spinal accessory nerves, must be meticulously preserved. Therefore, the discomfort experienced by surgeons during endoscopic thyroid surgery and concerns regarding surgical stability should be understood in a context slightly different from the discomfort encountered during other minimally invasive operations, including abdominal or thoracic surgery.

Surgical robots have not been developed specifically for thyroid surgeries. However, their use in thyroid surgery has provided tremendous benefits to surgeons and patients, and their utilization continues to increase. Among these methods, robotic transaxillary thyroid surgery (RTTS), first performed at Severance Hospital in 2007, is the most widely performed surgery worldwide, including in Korea, the United States, and Europe^14–17^. In April 2023, our institution achieved a milestone of 10,000 robotic thyroid surgeries, which is a record-breaking accomplishment. In this article, we discuss our experience of performing 10,000 cases of RTTS, with a particular focus on expanding the indications for robotic thyroid surgery and the stability of outcomes.

## Methods

### Patients

Between October 2007 and April 2023, 10,000 patients underwent RTTS at the Department of Surgery of the Yonsei University Health System. Until December 2018, four-arm-based robots, including the da Vinci S, Si, and Xi surgical robotic systems (Intuitive Surgical), were used. The da Vinci SP surgical robotic system (Intuitive Surgical) was introduced thereafter, and both systems are currently used in RTTS.

The decision regarding the necessity of surgery for thyroid nodules or benign thyroid diseases, including Graves’ disease, was based on the American Thyroid Association (ATA) guidelines^18,19^. Thyroid nodules were identified preoperatively using ultrasonography-guided fine-needle aspiration. Among patients with Graves’ disease, those who exhibited medical treatment resistance and had contraindications to radioactive iodine (RAI) therapy, those with severe exophthalmos, and those planning to conceive within the next year were included. All patients opted for the robotic procedure based on their preferences rather than undergoing open surgery. There were no contraindications for robotic surgery based on the type of thyroid cancer. The eligibility criteria for robotic surgery are described later.

Details of the clinicopathological characteristics, operative times, perioperative complications, and oncological outcomes were reviewed retrospectively. This study was conducted in accordance with the tenets of the Declaration of Helsinki (as revised in 2013) and was approved by the institutional review board of Yonsei University (IRB no.: 4-2023-0304). The requirement for informed consent was waived by the institutional review board of Yonsei University owing to the retrospective nature of this study.

### Operative method

In previous publications, the methodologies for both RTTS were described, including robotic modified radical neck dissection (MRND)^4,12–14,20–22^. Initially, two incisions were used for RTTS: one in the axilla and the other in front of the chest. These two incisions have been revised to use a single incision in the axilla since September 2009 for RT and March 2013 for robotic MRND.

In this method, the surgeon dissects the flap from the axilla to the thyroid in the operative field to expose the thyroid before attaching it to the robot. This method is called conventional RTTS. However, since the introduction of da Vinci SP in 2018, it has been used in conjunction with conventional RTTS and single-port transaxillary robotic thyroidectomy with a two-step retraction method^23^.

### Surgical extent

Surgical extent included less than total thyroidectomy (LTT), bilateral total thyroidectomy (BTT), MRND, and completion of thyroidectomy (CTT). LTT was defined as the total resection of one lobe of the thyroid with or without the removal of the nodule of the opposite lobe alone. Partial or subtotal thyroidectomies were performed only in cases in which the nodule on the opposite side had a small size and benign appearance, such as a purely cystic feature, or when a preoperative biopsy of the nodule confirmed Bethesda category II, and most importantly, when patients expressed a desire for its removal. All the patients with thyroid cancer underwent prophylactic or therapeutic ipsilateral central compartment neck dissection (CCND). Both MRND and CCND were performed using systematic microdissection or compartment dissection.

### Surgeon education and information

All surgeries were conducted using a team-based approach involving senior and junior surgeons. The junior surgeon was typically a clinical instructor with over a year of experience in open thyroidectomy or an international fellow. The pace of learning varies for each individual; however, typically, approximately 1–3 months after exposure to robotic surgery, individuals begin to learn the robotic technique under the guidance of a senior surgeon. During this period, they attempt to operate the console under supervision until they reach a stage of independence. Trainees with less than 3 months of robotic console training, even when participating in total thyroidectomy cases, are limited to performing only ipsilateral thyroidectomy. The senior surgeon handles the contralateral thyroidectomy, providing education and training gradually.

### Postoperative management and assessment

Each patient was equipped with a drain to mitigate the risk of postoperative seroma or hematoma. The patient was discharged after drain removal. Following surgery, levothyroxine was administered to patients post-RTTS, necessitating thyroid function maintenance or thyroid-stimulating hormone suppression for malignancy. Patients who underwent total thyroidectomy and met the indications for RAI ablation according to the ATA guidelines received ablation treatment. Subsequently, the patients underwent post-therapy whole-body and diagnostic whole-body scans. Following RAI treatment, stimulated serum thyroglobulin (Tg) levels were assessed 3 months, 1 year, and 5 years after the initial surgery. Suppressed serum Tg levels were measured annually to ensure oncological safety. Hypoparathyroidism and RLN palsy were classified as permanent complications if they were unresolved within 6 months.

To assess long-term cancer outcomes, we checked suppressed serum Tg levels annually and conducted regular ultrasound follow-ups for all patients. Those who exhibited recurrence or distant metastasis were subjected to additional imaging techniques, such as neck computed tomography (CT) or positron emission tomography-CT. Ultrasound-guided fine-needle aspiration biopsy was performed if neck node recurrence was suspected.

### Review of patients beyond previous indications

In our study published in 2018^24^, which documented 5000 cases of robotic thyroid surgery, the eligibility criteria for RTTS were: (1) follicular proliferation with a tumor size < 5 cm and (2) differentiated thyroid cancer without contraindications to RTTS. Since the contraindications for RTTS, including (1) previous head and neck surgery or irradiation, (2) definite tumor invasion to an adjacent organ (RLN, esophagus, or trachea), (3) multiple lateral neck node metastases or perinodal metastatic lymph node infiltration, and (4) distant metastasis, have remained consistent, we reviewed patients with a tumor size of 5 cm or larger based on their final pathology and those with Graves’ disease^25^, a representative goiter condition. The review included surgical records, postoperative histopathological findings, complications, and current status.

### Study endpoints

The primary endpoint of the study was to review the clinicopathological characteristics, surgical/oncological outcomes, and postoperative complications in 10,000 patients who underwent RTTS. The secondary endpoint involved a broader redefinition of the eligibility criteria for RTTS established in our previous study.

## Results

Of the 10,000 patients, 9461 (94.0%) had thyroid cancer, and 539 (5.4%) had benign thyroid nodules or Graves’ disease (Table 1). Four-arm-based robots were used for 8408 (84.1%) cases, and 1592 (15.9%) cases were performed with the da Vinci SP surgical robotic system. LTT was performed in 6870 patients (68.7%), and BTT was performed in 2305 (23.1%). CTT was performed in 45 patients (0.5%), and robotic MRND with or without thyroidectomy was performed in 780 patients (7.8%) with lateral neck node metastases. The operative time for RTTS averaged 131.9 ± 58.4 min (range 45–635), and the average postoperative hospital stay duration was 3.4 ± 0.6 days (range 2–24).

**Table 1:** Surgical information of 10,000 patients.

Variables	N = 10,000
Age (years)	37.81 ± 9.94 (range 8–77)
Sex ratio, M:F (F%)	1049:8951 (89.5)
Robotic platform, n (%)	
Four-arm-based robot (da Vinci S, Si, Xi)	8408 (84.1)
da Vinci SP	1592 (15.9)
Pathology, n (%)	
Malignancy	9461 (94.0)
Benign	539 (54.0)
Surgical extent, n (%)	
Less than total thyroidectomy	6870 (68.7)
Bilateral total thyroidectomy	2305 (23.1)
Completion of total thyroidectomy	45 (0.5)
MRND	780 (7.8)
Operative time (min)	131.9 ± 58.4 (range 45–635)
Postoperative hospital stay (days)	3.4 ± 0.6 (range 2–24)

*N* number, *MRND* modified radical neck dissection.

Table 2 presents detailed information on the patients with thyroid cancer. Among the 9461 patients who underwent RTTS and were diagnosed with thyroid cancer, 9348 (98.8%) were diagnosed with papillary thyroid carcinoma, 82 (0.9%) with follicular thyroid carcinoma, 13 (0.1%) with oncocytic carcinoma, 16 (0.2%) with medullary thyroid carcinoma, and 3 (0.0%) with poorly differentiated thyroid carcinoma. Of the 9461 patients, 9367 (99.0%) underwent initial surgery for thyroid cancer, whereas 94 (1.0%) underwent repeat surgery due to recurrence after their initial surgery. Among the 9367 patients who underwent the first surgery, the mean tumor size was 0.9 ± 0.7 (range 0.01–8.60). The retrieved central and positive central lymph nodes were 5.11 ± 3.95 (0–40) and 1.17 ± 2.11 (0–27), respectively. The retrieved lateral and positive lateral lymph nodes were 36.02 ± 17.46 (8–146) and 5.18 ± 4.02 (1–34), respectively. In 117 cases (1.2%), RLN was sacrificed, or the RLN, trachea, or esophagus was shaved due to T4 status. In addition, 12 patients (0.1%) were classified as M1. Of the 9367 patients who underwent initial surgery for thyroid cancer, recurrence occurred in 100 (1.1%).

**Table 2:** Detailed information on patients with thyroid cancer.

Variables	
Total patients with malignancy	N = 9461
Pathology, n (%)	
(PTC/FTC/OTC/MTC/PDTC)	9348 (98.8)/82 (0.9)/13 (0.1)/16 (0.2)/3 (0.0)
Initial vs. re-do operation, n (%)	9367 (99.0):94 (1.0)
Surgical extent, n (%)	
Less than total thyroidectomy	6435 (68.0)
Bilateral total thyroidectomy	2201 (23.3)
Completion of total thyroidectomy	45 (0.5)
Immediate completion for RAIT	6
Completion d/t recurrence	39
MRND	780 (8.2)
BTT + MRND	731
Unilateral MRND	49
Bilateral MRND	40
Completion total with MRND	16
MRND only, etc	33
Patients with the initial operation	N = 9367
Surgical extent, n (%)	
Less than total thyroidectomy	6435 (68.7)
BTT	2201 (23.5)
BTT + MRND (unilateral)	691 (7.4)
BTT + MRND (both)	40 (0.4)
Tumor size (cm)	0.9 ± 0.7 (range, 0.01–8.60)
Number of retrieved LNs (n)	
Central LN (retrieved/positive)	5.11 ± 3.95 (0–40)/1.17 ± 2.11 (0–27)
Lateral LN (retrieved/positive)	36.02 ± 17.46 (8–146)/5.18 ± 4.02 (1–34)
T classification (T1/T2/T3/T4), n (%)	6210 (66.3)/233 (2.5)/2807 (30.0)/117 (1.2)
N classification (N0/N1a/N1b), n (%)	5516 (58.9)/3120 (33.3)/731 (7.8)
M classification (M0/M1), n (%)	9355 (99.9): 12 (0.1)
Extrathyroidal extension (+), n (%)	2713 (29.0)
Multifocality (+), n (%)	1170 (12.5)
Bilaterality (+), n (%)	1238 (13.2)
Recurrence rate, n (%)	100 (1.1)

*PTC* papillary thyroid carcinoma, *FTC* follicular thyroid carcinoma, *OTC* oncocytic thyroid carcinoma, *MTC* medullary thyroid carcinoma, *PDTC* poorly differentiated thyroid carcinoma, *RAIT* radioactive iodine therapy, *BTT* bilateral total thyroidectomy, *MRND* modified radical neck dissection, *LN* lymph node.

Table 3 presents information on 539 patients who underwent RTTS and were diagnosed with benign thyroid tumors or Graves’ disease in the final pathology. Among the patients with existing tumors, the mean tumor size was 2.4 ± 1.4 (range 0.2–6.0) cm, and 97 patients (18.0%) underwent surgery for Graves’ disease.

**Table 3:** Pathological characteristics of patients with benign thyroid tumors or Graves’ disease.

Variables	Benign tumors N = 442	Graves’ disease N = 97
Mean tumor size (cm)	2.4 ± 1.4 (range 0.2–6.0)	
Pathology, n (%)		
Adenomatous hyperplasia	241 (54.5)	
Follicular adenoma	120 (27.1)	
Hürthle cell adenoma	31 (7.0)	
Lymphocytic thyroiditis	13 (2.9)	
NIFTP	26 (5.9)	
DH with LI		97 (100)
Others	11 (2.5)	
Surgical extent, n (%)		
Less than total thyroidectomy	421 (95.2)	14 (14.4)
Bilateral total thyroidectomy	21 (4.8)	83 (85.6)

*N* number, *NIFTP* non-invasive follicular thyroid neoplasm, *DH* diffuse hyperplasia, *LI* lymphocytic infiltration, *NTT* near-total thyroidectomy.

**Figure 1:**
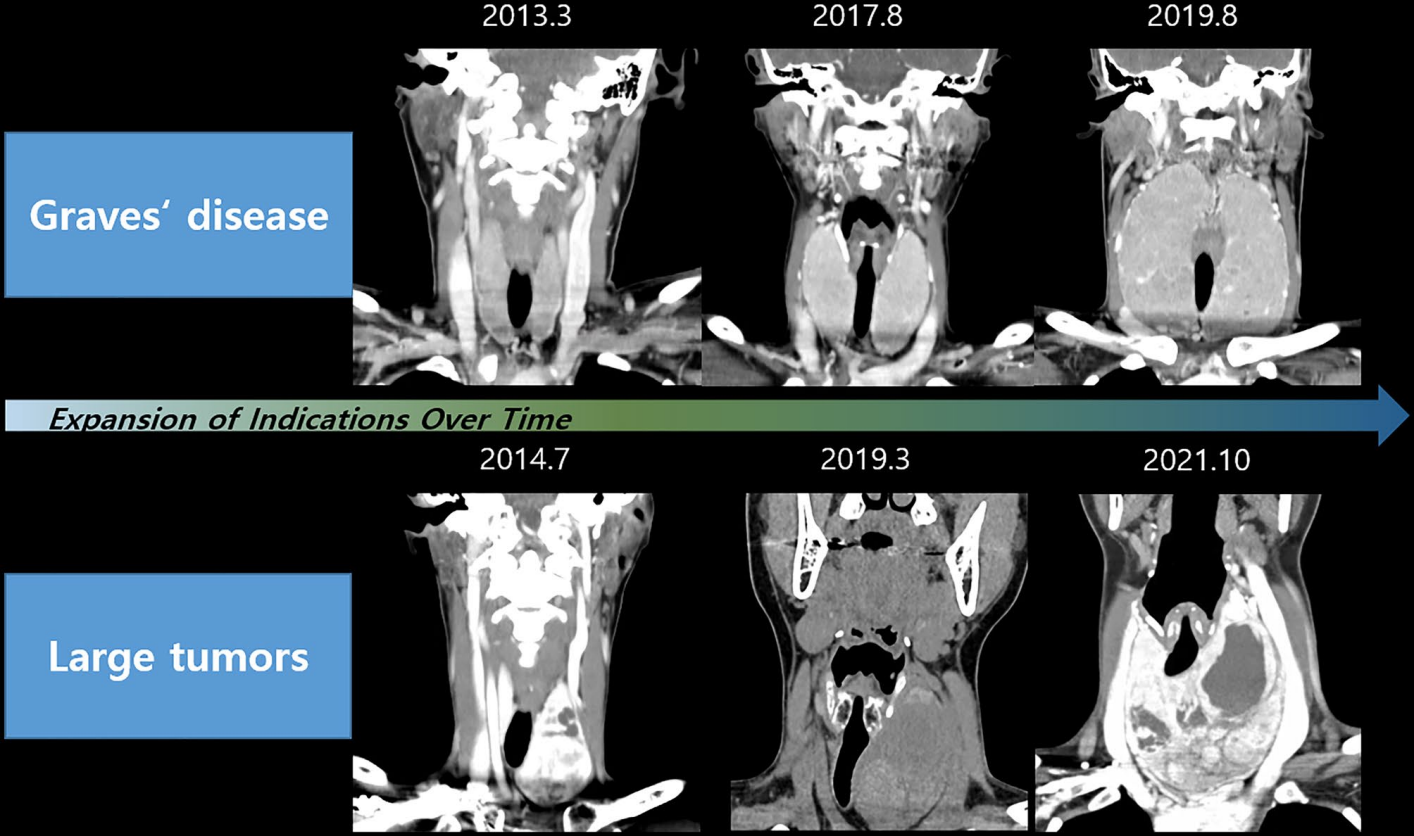
Progressive expansion of the indications for RTTS over time. *RTTS* robotic transaxillary thyroid surgery.

We analyzed the clinicopathological characteristics of 53 patients diagnosed with tumors larger than 5 cm based on their final pathology (Table 4). The mean age of these individuals was 35.2 ± 13.1 (range 8–69) years, with women comprising 46 (86.8%) of the cases. In addition, 12 patients (22.6%) were evaluated using the da Vinci SP system. The final pathology revealed malignancy in 29 (54.7%) cases and benign findings in 24 (45.3%) cases. The mean tumor size was 5.4 ± 0.6 (range 5.0–8.6), and the mean operative time was 141.4 ± 50.1 (range 73–291) min. Among these, two cases involved bilateral total thyroidectomy and right MRND. Only one patient exhibited transient hypoparathyroidism as a complication. We depicted representative cases of large nodules and Graves’ disease over time, illustrating the expansion of indications for RTTS (Fig. 1) using neck CT images. Among these cases, a surgical video from October 2021, with a procedure duration of 167 min, was included as a Supplementary Video 1.

**Table 4:** Clinicopathological characteristics of patients with tumors ≥ 5 cm (n = 53).

Variables	N = 53
Age (years)	35.2 ± 13.1 (range 8–69)
Sex ratio, M:F (F%)	7:46 (86.8)
Robotic platform, n (%)	
Four-arm-based robot (da Vinci S, Si, Xi)	41 (77.4)
da Vinci SP	12 (22.6)
Pathology, n (%)	
Malignancy	29 (54.7)
Benign	24 (45.3)
Tumor size (cm)	5.4 ± 0.6 (range 5.0–8.6)
Surgical extent, n (%)	
Less than total thyroidectomy	42 (79.2)
Bilateral total thyroidectomy	7 (13.2)
Completion total thyroidectomy	2 (3.8)
BTT + MRND	2 (3.8)
Operative time (min)	141.4 ± 50.1 (range 73–291)
Postoperative hospital stay (days)	3.2 ± 0.5 (range 2–5)
Postoperative complication	
Transient hypoparathyroidism, n (n/BTT, %)	1 (1/11, 9.1)

*N* number, *M* male, *F* female, *BTT* bilateral total thyroidectomy, *MRND* modified radical neck dissection.

Transient hypoparathyroidism was the most common postoperative complication (Table 5), occurring in 1189 of the 3136 patients (37.91%) who underwent total thyroidectomy. Among them, only 33 (1.52%) progressed to permanent hypoparathyroidism. Transient hoarseness occurred in 210 cases (2.1%), and RLN injury occurred in 32 cases (0.32%), with four cases involving cancer invasion requiring sacrifice. Seroma formation led to aspiration in 153 patients, whereas hematoma formation resulted in close observation or reoperation in 61 patients. In addition, 32 patients experienced prolonged hospitalization due to chyle leakage. A specific brachial plexus injury unique to RTTS, which does not occur in open surgery, was observed in three patients.

**Table 5:** Postoperative complications.

Variables	N = 10,000
Transient hypoparathyroidism (n/BTT, %)	1189 (1189/3136, 37.91%)
Permanent hypoparathyroidism (n/BTT, %)	33 (33/3136, 1.52%)
Transient hoarseness, n (%)	210 (2.1%)
Permanent RLN injury, n (%)	32 (0.32%)
RLN sacrifice d/t cancer invasion	4 (0.04%)
Seroma formation, n (%)	153 (1.53%)
Hematoma formation, n (%)	61 (0.61%)
Conservative management	36 (0.36%)
Reoperation	25 (0.25%)
Chyle leakage, n (%)	32 (4.10%)
Wound infection, n (%)	11 (0.11)
Horner’s syndrome, n (%)	4 (0.76%)
Vagus nerve injury	1 (0.12%)
Trachea injury, n (%)	9 (0.09)
Vessel injury (carotid artery, brachiocephalic vein), n (%)	2 (0.02)
Brachial plexus injury, n (%)	5 (0.05)
Spinal accessory nerve injury, n (%)	3 (0.03)
Flap injury (burn), n (%)	15 (0.15)

*N* number, *BTT* bilateral total thyroidectomy, *MRND* modified radical neck dissection, *LN* lymph node, *RLN* recurrent laryngeal nerve.

Table 6 presents the average off-Tg and on-Tg levels investigated in the 2,236 patients who received RAI among the 9367 patients who underwent the first surgery. The serum Tg levels after 3 months (Off-Tg), 1 year (On-Tg), and 5 years (On-Tg) were 8.5 ± 23.5 (< 0.1–384.6), 0.8 ± 5.2 (< 0.1–113.5), and 0.4 ± 1.4 (< 0.1–4.3), respectively. Among the 6435 patients who underwent LTT, 54 (0.8%) experienced recurrence, whereas among the 2932 patients who underwent BTT (± MRND), 46 (1.6%) exhibited signs of recurrence.

**Table 6:** Oncological outcomes of patients (s/p at initial surgery).

Variables	N = 9367	
Operation extent	LTT (n = 6435)	BTT (± MRND) (n = 2932)
Postoperative RAI ablation	–	2236
Serum Tg after 3 months (Off-Tg) (ng/mL)	–	8.5 ± 23.5 (< 0.1–384.6)
Serum Tg after 1 year (On-Tg) (ng/mL)	–	0.8 ± 5.2 (< 0.1–113.5)
Serum Tg after 5 years (On-Tg) (ng/mL)	–	0.4 ± 1.4 (< 0.1–4.3)
Recurrence	54 (0.8%)	46 (1.6%)

*N* number, *LTT* less than total thyroidectomy, *BTT* bilateral total thyroidectomy, *MRND* modified radical neck dissection, *RAI* radioactive iodine, *Tg* thyroglobulin.

*Twelve patients with distant metastasis were excluded from the Tg level calculations.

## Discussion

It took 15.5 years to achieve the milestone of 10,000 cases of robotic thyroid surgery^13^. During this period, not only were the surgical and oncological outcomes of RTTS found to be feasible and safe, but the scope of indications was also gradually expanded. To understand the reasons behind this phenomenon, various contextual features cannot be overlooked. The unique characteristics of thyroid diseases, including thyroid cancer, and the favorable medical landscape in Korea, which allows patients to freely choose RTTS, have played a vital role in this achievement. This study presents the insights gained from performing 10,000 cases of RTTS at a single institution, along with the expansion of indications over time.

The desire for the RTTS has considerably increased because of several factors. First, conventional open thyroidectomy leaves a visible scar on the anterior neck, drawing attention from others. Second, thyroid disorders, including thyroid cancer, are more prevalent in young women who are sensitive to aesthetics. Finally, the generally favorable prognosis of thyroid malignancies^26^, which often does not significantly impact overall health, further motivates the preference for robotic surgery and allows surgeons to conceal incisions in less visible areas of the body.

In the context of the South Korean healthcare system, the ability of patients to choose their preferred surgical approach plays a crucial role in eliminating selection bias concerning indications for RTTS. Similar to all surgical procedures, in the early stages of its introduction, RTTS was primarily performed in patients with a straightforward anatomy, where simple thyroidectomy was sufficient for treatment. However, as information about RTTS became more widely available, patients began expressing demand for such procedures. As our medical team became accustomed to RTTS, we realized that with increased proficiency, we could embrace more challenging cases. Most importantly, we recognized the significance of acknowledging patients’ desires for RTTS and respecting their preferences.

Even patients with large goiters or advanced cancer, highly muscular men, and those who experienced cancer recurrence expressed their desire for RTTS for various reasons, such as keloid-prone skin, professional obligations, or private reasons. Despite being cautioned about the potential risks of open conversion or blood transfusion, these patients still chose to undergo RTTS. The requests for those seeking a better quality of life are rooted in the indolent nature of thyroid cancer, which does not immediately threaten one’s life and the condition of being afflicted by a disease at a young age with a long life expectancy.

For those beginning robot-assisted thyroid surgery, the contraindications presented by Kim et al. in the previous paper should be helpful: (1) previous head and neck surgery or irradiation; (2) definite tumor invasion to an adjacent organ (RLN, esophagus, or trachea); (3) multiple lateral neck node metastases or perinodal metastatic lymph node infiltration; and (4) distant metastasis. However, if this procedure is performed repeatedly, unexpected situations may arise. Based on our experience, the preoperative evaluation indicated the absence of invasion of surrounding organs. Yet, during surgery, situations arose where the sacrifice or shaving of RLN became necessary due to the direct invasion. Further, cases involving perinodal lymph node infiltration or cases requiring esophageal or tracheal shaving were encountered. In some instances, the patient opted for robotic surgery despite the diagnosis of lung metastasis before surgery. In other cases, lung metastases were detected during postoperative RAI therapy. Over time, we realized that these surgeries were not entirely unfeasible unless the cases required cooperation with other departments.

Recently, many papers on robotic thyroid surgery have been published, including 500 cases of lateral neck dissection^27^, surgeries for recurrent cancer^28^, and Graves’ disease^25^, and papers focusing on the utilization of the da Vinci SP system^23,29,30^. These publications can be viewed as evidence of the expansion of indications. This signifies that many surgeries previously conducted using conventional open approaches are now feasible using robots in almost all cases.

The long-term oncological outcomes of RTTS demonstrate that it is not a superficially emphasized temporary surgical approach merely for cosmetic purposes in treating thyroid cancer. The uniformity in the surgical content is attributed to the ability of the transaxillary approach to provide a working space and visibility comparable to those of conventional open surgery. Moreover, RTTS allows for the resolution of recurrent cancer, severe goiter, and postoperative bleeding through the transaxillary approach without the need for open conversion. Maintaining the embryological plane, the subplatysmal layer, as a surgical plane in RTTS, rather than using blunt insertion of trocars, leads to reduced adhesion during reoperation and facilitates the easy removal of goiter specimens or hematomas through the axillary incision.

One of the challenges that RTTS had to overcome was the wide range of flap dissections required, which extended from the axilla to the thyroid gland. These issues have been considerably addressed using da Vinci SP^23,30^. The emergence of da Vinci SP allows surgeons to adjust the minimal range of the flap required for a specific surgery based on factors such as a patient’s physique or thyroid size. This can be interpreted in a similar context as adjusting the size of the incision or flap in conventional open thyroid surgery.

Ironically, in the case of a large goiter, some patients are willing to accept a wider flap extending from the axilla to the neck, considering that the scar will be less noticeable than if the surgery is performed directly on the neck. From the perspective of preserving the condition of the specimen during its removal from the human body, focusing solely on minimizing the flap size in patients with expected larger specimens could be considered impractical. Therefore, this aspect must be adequately explained to patients before surgery.

In the future, surgery can be performed using robots equipped with artificial intelligence. From this perspective, the potential for utilizing the transaxillary approach, which follows the embryological plane and provides adequate space and visibility tailored to each individual’s condition, is boundless. Although the absence of a randomized controlled study for RTTS can be considered a limitation, we have realized from achieving 10,000 cases that the surgical robot does not pose harm to patients, and skilled robotic surgeons can achieve much more than initially anticipated.

This study has some limitations owing to the heterogeneity of patients who underwent RTTS, which prevented statistical analysis. However, the outcomes we demonstrated with RTTS compare favorably with the classical references for open thyroid surgery in terms of oncological outcomes and complications. Complications of brachial plexus palsy, which were initially considered specific to RTTS, have been closely monitored since the early stages of RTTS development and no longer occur. One limitation in evaluating complications is that we did not conduct pre- or postoperative laryngoscopy in all patients. As only one-third of patients with vocal cord paralysis experience voice changes^31^, the occurrence of temporary or permanent vocal cord paralysis may have been underestimated in this study.

This report confirms that certain surgeries that were previously feasible only through open procedures can now be performed using robotic techniques. In conclusion, RTTS seems safe and feasible from both surgical and oncological perspectives, and the spectrum of indications suitable for RTTS surgery is progressively expanding.

### Supplementary Information

Supplementary Legends.

Supplementary Video 1.

## Data Availability

The data are available, but due to the potential risk of breaching individual privacy, access is restricted only to specific researchers, as approved by the Institutional Review Board (IRB). Dr. Jin Kyong Kim (jkkim3986@yuhs.ac) should be contacted if someone wants to request the data from this study.
