# Supplementary material for: Expansion of thyroid surgical territory through 10,000 cases under the da Vinci robotic knife
Source: Sci Rep. 2024 Mar 30;14:7555. doi: 10.1038/s41598-024-57163-2 (PMC10981764; doi:10.1038/s41598-024-57163-2)
Supplement: Supplementary file 1 — Supplementary Legends. [file 41598_2024_57163_MOESM1_ESM.docx]

**Legend for Supplementary Video**

**Supplementary video 1**. In October 2021, a successful robotic left total thyroidectomy with central compartment neck dissection was performed on a 23-year-old female patient who had an 8.6 cm-sized left thyroid nodule
